# The prevalence of autism in cerebellar malformations: a systematic review and meta-analysis

**DOI:** 10.1186/s11689-026-09691-3

**Published:** 2026-04-14

**Authors:** Douglas A. Wells, Danny Jaber, Shreya Dey, J. Seth VanZant, Ross A. Carson, Mary Lou Klem, Sima Sharghi, Ronald R. Seese

**Affiliations:** 1 Department of Neurology and Developmental Neurosciences, Baylor College of Medicine, Houston, TX USA; 2 Department of Pediatrics, Northeast Ohio Medical University, Rootstown, OH 44272 USA; 3 Department of Pediatrics, University of Pittsburgh School of Medicine, Pittsburgh, PA USA; 4 Health Science Library System, University of Pittsburgh, Pittsburgh, PA USA; 5 Rebecca D. Considine Research Institute, Akron Children’s Hospital, Akron, OH USA; 6 Department of Family and Community Medicine, Northeast Ohio Medical University, Rootstown, OH USA; 7 Department of Biomedical Sciences, Northeast Ohio Medical University, Rootstown, OH USA; 8 Department of Psychiatry, Northeast Ohio Medical University, Rootstown, OH USA

**Keywords:** Autism, Autistic, Cerebellum, Posterior fossa, Neuropsychiatry

## Abstract

The cerebellum contributes to the control of movement, language, and sociability. Purkinje cells, the main output neurons of the cerebellar cortex, are reduced in cerebellar malformations (CMs) and in autism spectrum disorder (ASD). Thus, CMs may increase the risk for ASD. We conducted a meta-analysis to determine the prevalence of ASD in individuals with CMs. We searched PubMed, Embase, and APA PsycINFO for studies assessing ASD in patients with radiologically confirmed CMs. We evaluated risk of bias using Joanna Briggs Institute Checklists. Finally, we extracted data to calculate ASD prevalence and assess variation across CM subtypes. From 1,564 screened articles, 32 studies met inclusion criteria, encompassing 1,032 individuals with CMs. ASD prevalence was 31.2%, significantly higher than the general population rate (1 in 31). High heterogeneity was observed (*I*² = 72.3%; *p* < 0.0001). Vermis and cerebellar hypoplasia reliably contributed to the high ASD prevalence. Many studies had small sample sizes and used different metrics to diagnose autism. ASD is more common in individuals with CMs, especially vermis malformations, than in the general population. Early neurodevelopmental monitoring may benefit those with prenatally diagnosed CMs.

**Supplementary Information:**

The online version contains supplementary material available at 10.1186/s11689-026-09691-3.

## Introduction

The cerebellum coordinates movement. This “little brain” also influences language, cognition, emotions, and sociability [1–3]. Schmahmann’s universal cerebellar transform theory proposes the cerebellum applies the same error-correcting operation across motor, cognitive, and affective domains [3]. Disruption of specific cerebellar circuits produces the cerebellar cognitive affective syndrome, including deficits in executive function and affect [3, 4]. Autism spectrum disorder (ASD) is a prevalent (1:31) neurodevelopmental disability characterized by social communication differences and restricted interests and behaviors [5, 6]. Cerebellar contributions to cognition, sociability, and emotion provide a theoretical basis for cerebellar involvement in ASD. This theoretical basis is supported by anatomical evidence. The cerebellum forms bidirectional closed-loop connections with motor and prefrontal cortices [1, 7, 8]. This closed-loop organization supports the possibility that cerebellar dysfunction contributes to the complex motor and behavioral phenotypes of ASD.

Clinical data from autistic individuals^1^ further support cerebellar involvement in autism. Neuroimaging data implicate the cerebellum in the pathophysiology of ASD [11, 12]. Sizes of cerebellar regions are decreased in ASD [13], including the vermis [14]. Purkinje cells are the neurons that project from the cerebellar cortex to influence output [15, 16]. Neuropathological data show reduced numbers of Purkinje cells in the cerebellums of autistic individuals compared to neurotypical individuals [17]. Changes in cerebellar function early in neurodevelopment result in social abnormalities [18]. These findings suggest cerebellar malformations (CMs) may increase the risk for ASD.

Cerebellar malformations (CMs) are developmental abnormalities often incidentally diagnosed during prenatal ultrasonography [19]. Prenatal diagnosis of CMs coupled with postnatal neurodevelopmental surveillance testing may improve outcomes in children with CMs and autism.

Intervention before 30 months of age for autistic children improves outcomes, including language, social communication, and adaptive behaviors [20–22]. Notably, children who begin therapy at 18 months demonstrate greater gains than those who start at 27 months, especially in language, social communication, and self-help skills [23]. An earlier diagnosis of ASD contributes to increased parental acceptance and satisfaction with the diagnostic process, while reducing parental stress [24]. Early intervention is associated with decreased healthcare expenditures by minimizing the need for more intensive and prolonged services [25]. Children diagnosed before 36 months of age were more likely to attend mainstream classes and require less support [26]. Given these benefits of early intervention in other causes of ASD, infants diagnosed in utero with a CM may benefit from early surveillance for ASD during early childhood. With timely diagnosis, patients and families may benefit from therapy-based interventions, individualized education plans, and social skills programs.

To begin to understand how ASD is associated with CMs, we estimated the prevalence of ASD in individuals with CMs. We performed a systematic review of the literature and completed a meta-analysis of the resulting data. Our primary objective was to estimate the prevalence of ASD among those with CMs. Our secondary objective was to determine how different subtypes of CMs influence ASD prevalence.

## Methods

The Preferred Reporting Items for Systematic Review and Meta-Analysis (PRISMA) Statement guided the reporting of this study [27].

### Information sources and search strategy

We searched PubMed (National Library of Medicine), Embase (Elsevier), and APA PsycINFO (Ovid) databases for references. We designed a PubMed search using Medical Subject Headings and natural language terms to create concept clusters representing “cerebellar malformations” and “neurodevelopmental disorders” (see Supplemental Information Table 1). We validated the search by testing its ability to retrieve a known set of records. We translated the search for use in Embase and APA PsycINFO. We ran searches with an English language limit on July 7, 2020. We downloaded the results to EndNote (Version X9, Clarivate Analytics) and removed duplicates [28]. We uploaded the remaining records to DistillerSR (Version 2023.8.2, DistillerSR Inc., 2024).

### Eligibility and exclusionary criteria

Epidemiological studies, randomized control trials, observational studies (e.g., cross-sectional, case-control, and cohort studies), and case series that included patients with radiologically confirmed CMs were eligible to be analyzed. We defined CMs as vermis hypoplasia, Joubert syndrome, rhombencephalosynapsis, Dandy-Walker syndrome, complete cerebellar agenesis, cerebellar hypoplasia, mega cisterna magna, Chiari I malformation, cerebellar cyst, and cerebellar hemisphere abnormalities.

We excluded non-English studies, case reports, editorials, commentaries, letters to editors, author replies, animal studies, and studies that did not report a diagnosis of ASD. Case reports were excluded because we could not generate prevalence data from a sample size of one.

### Study selection

Two independent reviewers conducted a three-tier selection process. For any disagreement, we recruited a third reviewer and reached consensus through discussion.

In tier one, we assessed titles and abstracts. First, we determined if an article was published in a language other than English or included only one patient. Second, we evaluated if the article failed to address the presence of any neurodevelopmental disability (NDD) and/or did not mention CMs. We excluded articles that met these criteria.

In tier two, we assessed titles, abstracts, and full texts of articles that passed tier one. First, we determined if an article failed to radiologically confirm a CM. Second, we assessed if an article only included a single person with a CM or if the article did not comment on the presence of any NDD. If any of these scenarios were true, we excluded the articles.

In tier three, we assessed full texts of articles that passed tier two. First, we determined if participants were younger than 16 months old because ASD screening generally starts at this age [29]. Second, we determined if the sample size was one. Third, we determined if the study included cerebellar size but did not diagnose a CM. If any of these scenarios were true, we excluded the study.

### Risk of bias assessment

**Fig. 1:**
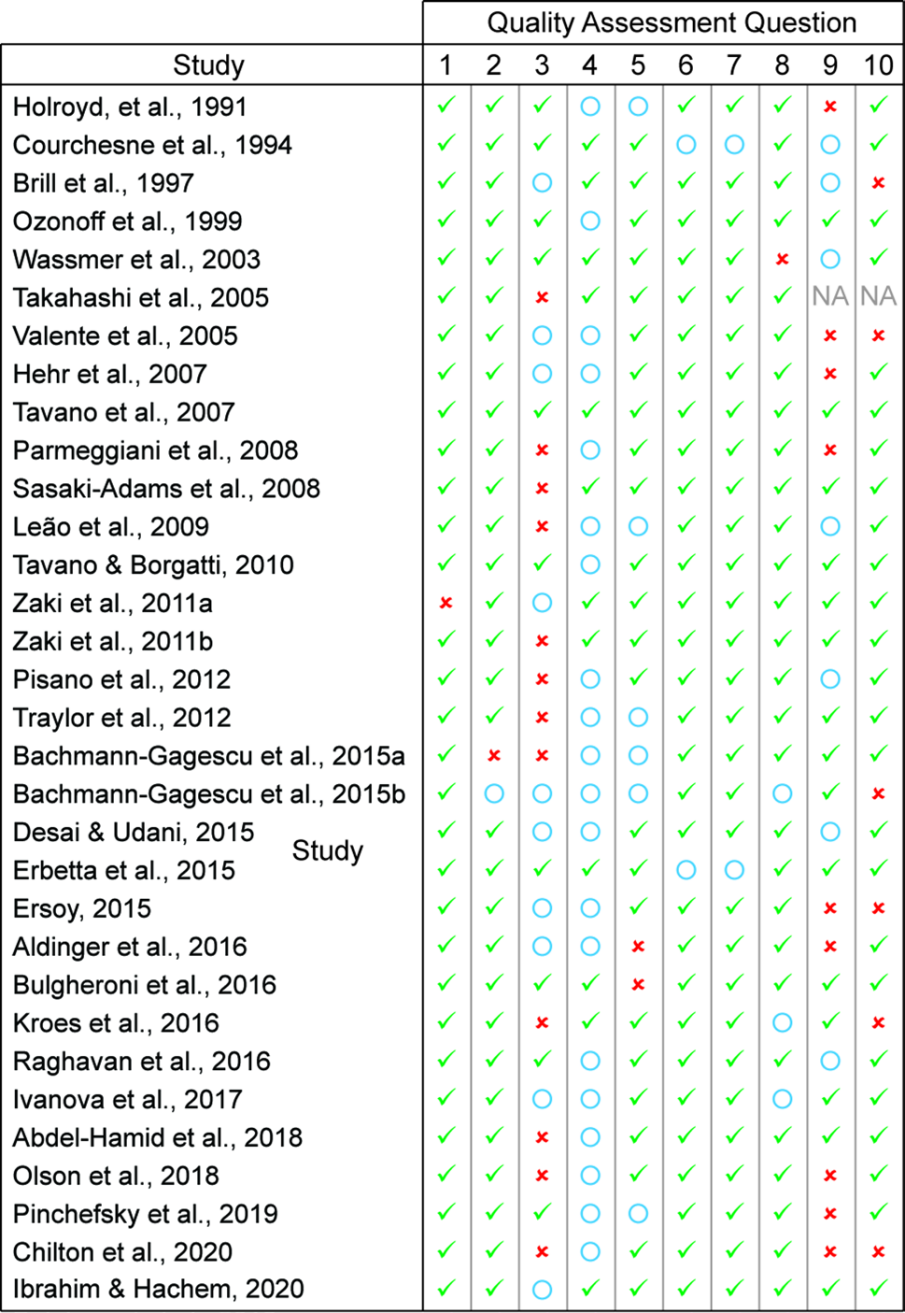
Risk of bias assessments with the JBI checklists. We completed JBI checklists for all articles that made it through screening. Highest risk of bias was associated with “No” answers (red x). Medium risk of bias was associated with “Unclear” answers (blue circle). Lowest risk of bias was associated with “Yes” answers (green check). Please see Supplemental Information Table 2 for lists of the questions used in the JBI checklists employed in this study

Two reviewers assessed each study’s risk of bias using the Joanna Briggs Institute (JBI) Critical Appraisal Tools (Fig. 1). We implemented 10-domain checklists for case-series and case-control articles and an 8-domain checklist for cross-sectional studies (Supplemental Information Table 2). We resolved discordance by discussion. If the highest risk of bias was present in more than 50% of the domains, a study was excluded from data extraction.

### Data extraction

Two reviewers independently extracted data from the 32 articles. If discordance occurred, we reached consensus through discussion or by using a third reviewer. The reviewers extracted multiple variables (Table 1), including total number of subjects with any CM, number of subjects with each specific CM, total number of subjects diagnosed with ASD (regardless of any reported co-morbidities and/or genetic variants), number of subjects with ASD in each specific CM, and psychometric test used to diagnose ASD (when applicable). When clarification on any of the above information was needed, we contacted the article’s corresponding author. Because primary sources did not consistently report patient sex, age at ASD diagnosis, co-morbid medical diagnoses, or genetic variants, we did not extract this data.

**Table 1:**
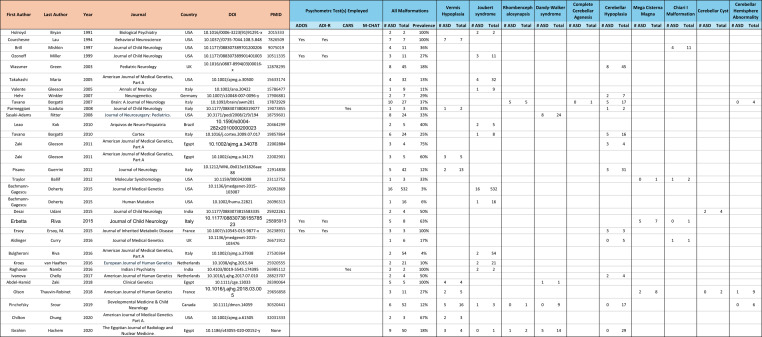
Extracted data from included records

### Statistical analyses

We used RStudio (version 4.4.1; R Foundation for Statistical Computing) to complete the meta-analyses. Study proportions were logit-transformed and pooled using a random effects model. Fixed effects results were also reported for comparison. For individual studies, we used Clopper-Pearson exact CIs, with a continuity correction of 0.5 when needed. Between-study heterogeneity was quantified with Cochran’s *Q*, *τ²*, *I²*, and *H*. To define sources of heterogeneity, we fit mixed-effects meta-regression models with study-level binary indicators to test whether specific CM subtypes were represented in the cohort. We assessed robustness with leave-one-out analyses and influence diagnostics. We evaluated publication bias with a funnel plot and a Duval-Tweedie trim-and-fill approach.

## Results

### Included studies and sample characteristics

**Fig. 2:**
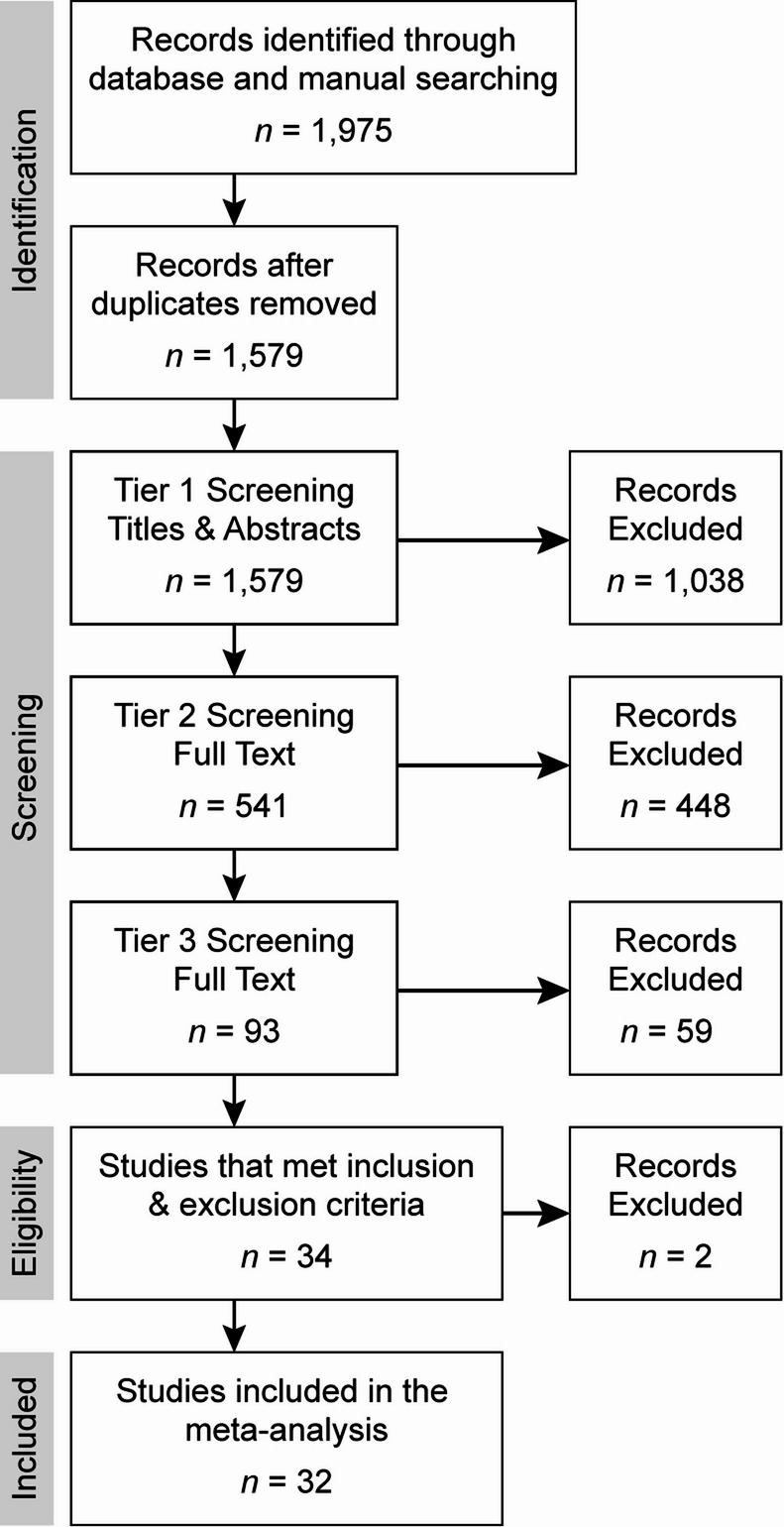
PRISMA flow diagram of study selection. We identified 1,975 articles and removed 396 duplicates. We used a three-tiered screening approach that identified 34 articles that met our study’s inclusion and exclusion criteria. We excluded two records in which patients were reported by the same authors in other records. We completed meta-analyses on the resultant 32 records

We identified 1,975 records (Fig. 2) and removed 396 duplicates to analyze 1,579 records.

We completed a three-tier screening process. After screening the titles and abstracts of the 1,579 records in tier one, we excluded 1,038 articles. From the resulting 541 articles, we screened the full texts against inclusion and exclusion criteria. In tier two, we excluded 507 studies. In tier three, we reviewed all aspects of the articles and excluded 59 studies. This work resulted in 34 articles published between 1991 and 2020 that met inclusion criteria. We removed two articles because the patients were already included in other publications by the same authors. Of the resulting 32 studies, 28 were case series, three were case-control studies, and one was cross-sectional.

For these 32 studies, we completed a JBI Critical Appraisal Checklist to assess for the risk of bias (Fig. 1). For each article, the percentage of questions in the highest risk of bias category was 50% or less (11% ± 8%, mean ± standard deviation (SD)). As such, all 32 articles moved to the data extraction phase.

### Included subjects

Over these 32 articles, we analyzed 1,032 subjects. The subtype of CM that had the most subjects was Joubert syndrome (68% or 699 of 1,032 total subjects). Over 79% (812) of the 1,032 subjects had a CM that involved the vermis. A minority of studies (6 of 32 studies) utilized psychometric tool(s) to support a clinical diagnosis of ASD. This approach was used in 8.5% (88) of the 1,032 subjects across all studies. Otherwise, studies used clinician report to diagnose ASD or did not specify how a diagnosis of ASD was reached.

### Prevalence of ASD in CMs: meta-analysis

**Fig. 3:**
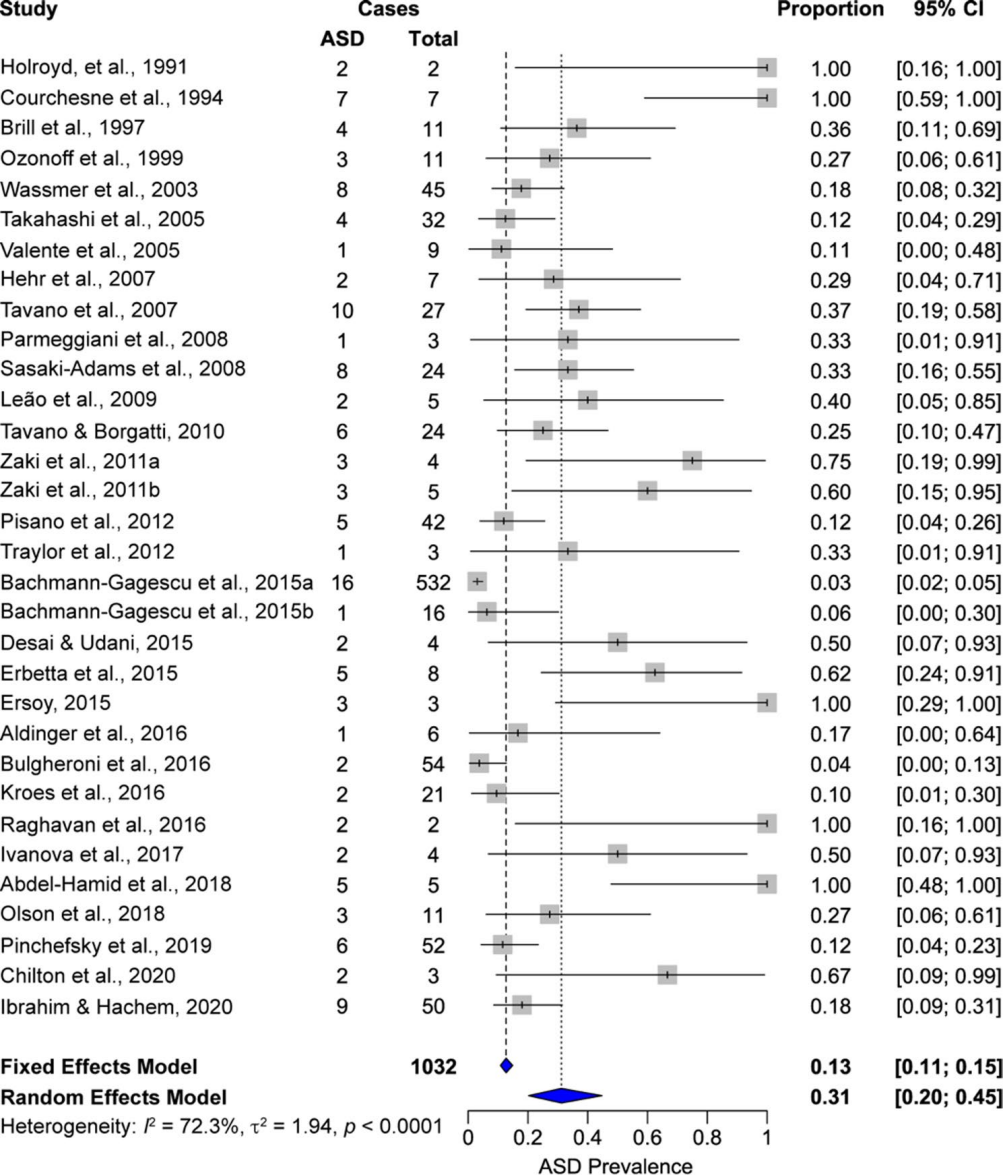
Forest plot of meta-analysis results. Pooled estimates of ASD prevalence in individuals with CMs. We sampled 1,032 subjects over 32 records. We calculated two pooled estimates, one from a fixed effects model (13%) and one from a random effects model (31%). We calculated heterogeneity to be high, supporting the use of the random effects model

As shown in the Forest Plot in Fig. 3, the pooled prevalence of ASD derived from the random effects model is 31.2% (95% confidence interval (CI): 20.2% to 44.7%). This result suggests that nearly one in three individuals was identified with ASD. The fixed effect model, which assumes the same prevalence across all studies, yielded a lower estimate of 12.7% (95% CI: 10.8% to 14.9%). This divergence between the two models supports the presence of heterogeneity.

To test this, we quantified heterogeneity. Cochran’s *Q* statistic was highly significant (*Q* = 111.9, df = 31, *p* = 4.06 × 10^− 16^). Thus, the observed variation in prevalence exceeds random chance alone. The *I*^*2*^ value was 72.3% (95% CI: 60.6% to 80.5%), suggesting that nearly three-quarters of the variability in prevalence is due to real differences rather than random error. The between-study variance was t^2^ = 1.94 (SD, t = 1.39) and the heterogeneity index was *H* = 1.90 (95% CI: 1.59 to 2.27). These results support the choice of a random effects model to estimate ASD prevalence.

**Fig. 4:**
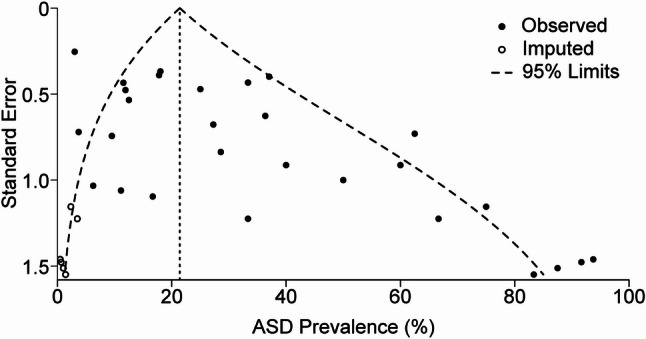
Assessment of publication bias. The trim and fill method uncovered seven studies on the left side of a funnel plot, suggesting that smaller studies reporting lower ASD prevalence are underrepresented in the literature. After including these potential studies, the pooled prevalence dropped from 31.2% to 21.7%. Open circles are imputed studies (k₀ = 7). Curved 95% pseudo-limits are shown. Dashed line indicates the trim-and-fill pooled prevalence (21.4%)

To test if publication bias contributed to the high heterogeneity, we completed a trim-and-fill analysis. The funnel plot was asymmetric (Fig. 4), indicating a lack of studies reporting lower ASD prevalence. Trim-and-fill analysis imputed 7 studies on the left and yielded an adjusted pooled prevalence of 21.4% (95% CI: 14.2% to 30.9%). Substantial heterogeneity persisted (*I*² = 77.5%, t² = 1.59; *Q* = 168.9, *p* < 0.0001), suggesting that asymmetry alone does not account for between-study differences.

To complement these pooled prevalence estimates, we calculated the arithmetic mean of ASD prevalence in CMs to be 40.9% (SD, 31.8%). This unweighted mean provides an overview of the prevalence distribution across studies but does not account for differences in sample size. The large SD indicates a high degree of variability and aligns with the heterogeneity in the meta-analysis, further supporting the use of a random effects model.

Finally, we calculated the median prevalence to be 33.3% with a wide interquartile range (IQR) of 45.0%: the 25th percentile (*Q*_*1*_) was 15.6% and the 75th percentile (*Q*_*3*_) was 60.6%. This spread within the middle 50% of the data confirms that heterogeneity is not limited to outlier studies. Rather, heterogeneity is present even in the central portion of the distribution. These data reinforce the justification for a random effects model that accommodates for differences in effect sizes rather than assuming a single common prevalence.

### Sensitivity analysis

**Fig. 5:**
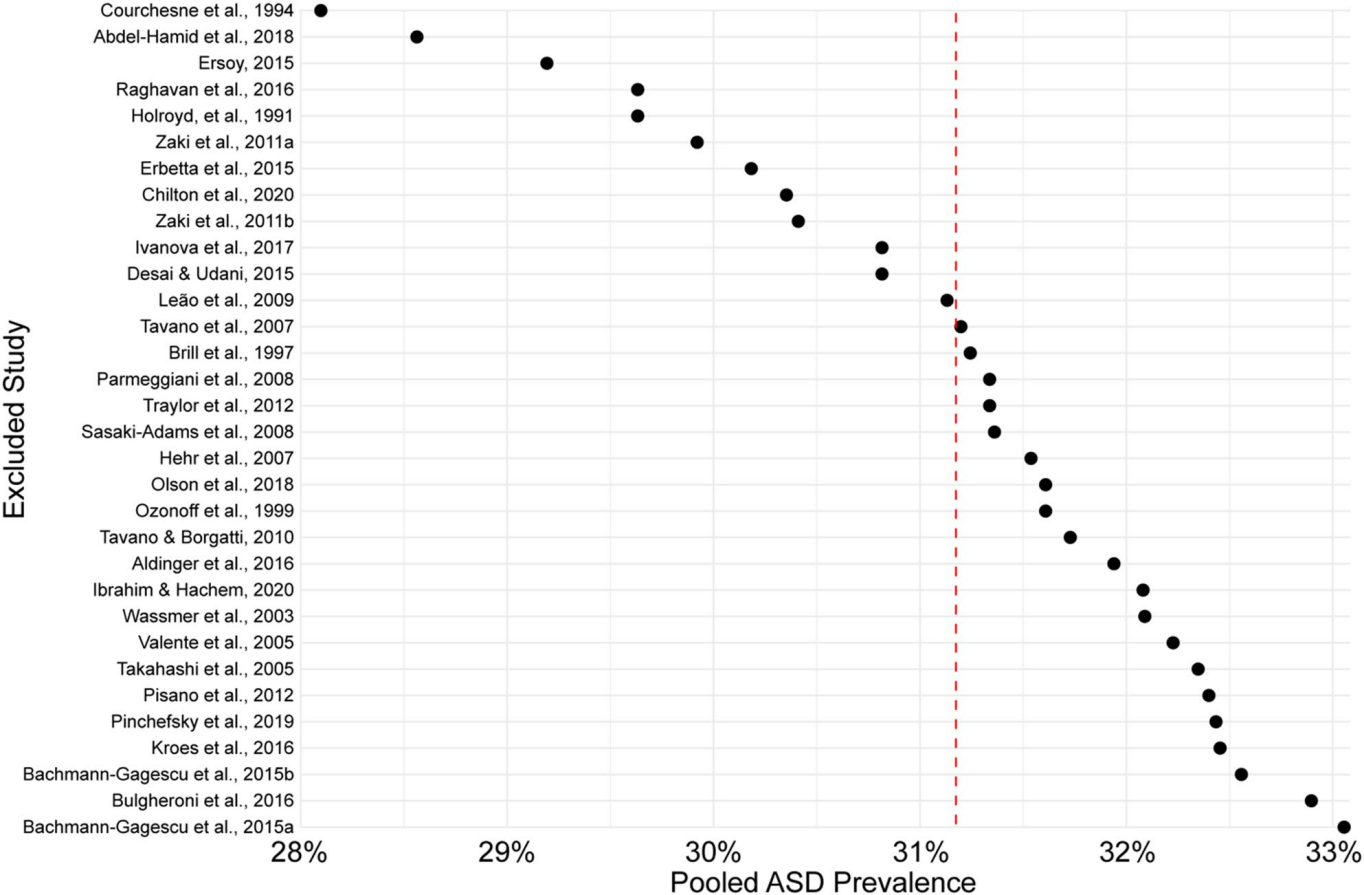
Leave-one-out sensitivity analysis. After removing one study at a time and calculating the pooled prevalence through a random effects model, we found that no single study exerted a disproportionate influence on our estimated ASD prevalence in CMs. Red dashed line indicates mean leave-one-out prevalence across the 32 analyses

We conducted a leave-one-out sensitivity analysis. We removed each study, one at a time, and recalculated the pooled ASD prevalence using a random effects model (Fig. 5). The logit-transformed pooled prevalence estimates across the 32 iterations ranged from − 0.9396 to -0.7057. When back-transformed to the original prevalence scale, this corresponds to 28.1% to 33.1%, with a mean leave-one-out prevalence of 31.2%. These data align with the random effects pooled estimate and suggest that no single study disproportionately influenced that estimate.

### Influence of specific CMs on ASD prevalence: meta-regression

To test if a specific malformation type caused a variation in ASD prevalence, we conducted subgroup analyses using meta-regression. The meta-regression examined the influence of Joubert syndrome, vermis hypoplasia, and cerebellar hypoplasia on ASD prevalence (Table 2). Other malformation types were not analyzed because of insufficient data to complete meta-regressions.

**Table 2:** Sub-group meta-regression analyses

Malformation	Coefficient	Confidence Interval	*P* value	Prevalence if Malformation Present	Prevalence if Malformation Not Present	*R* ^2^
Joubert Syndrome	-1.36	[-2.19, -0.53]	0.0014	38.7%	13.9%	38.8%
Vermis Hypoplasia	0.41	[-0.68, 1.50]	0.46	26.5%	35.2%	0%
Cerebellar Hypoplasia	0.31	[-0.76, 1.37]	0.57	26.8%	33.2%	0%

The meta-regression explaining the influence of Joubert syndrome on ASD prevalence included 32 studies. The model explained over one-third of the between-study heterogeneity (*R*^*2*^ = 38.8%). The test of moderators was statistically significant (*QM*(1) = 10.24, *p* = 0.0014), indicating that the presence of Joubert syndrome in a study significantly predicted ASD prevalence. The coefficient for the moderator was − 1.36 (95% CI: -2.19 to -0.53), suggesting that studies that included Joubert syndrome reported lower ASD prevalence than those that did not. When the intercept of the model (-0.46, *p* = 0.084) was back-transformed to the probability scale, the estimated ASD prevalence in studies with and without Joubert syndrome was 13.9% and 38.7%, respectively.

The meta-regression models explaining the influence of vermis hypoplasia and cerebellar hypoplasia on ASD prevalence included 32 studies. Neither model explained between-study heterogeneity (*R*^*2*^ = 0% for both). The tests of moderators were not statistically significant (vermis hypoplasia: *QM*(1) = 10.24, *p* = 0.4604; cerebellar hypoplasia: *QM*(1) = 0.32, *p* = 0.5732). Thus, presence of vermis hypoplasia or cerebellar hypoplasia in a study was not associated with differences in ASD prevalence compared to the meta-analysis results. The coefficients for the moderators were 0.41 (95% CI: -0.68 to 1.50) and 0.31 (95% CI: -0.76 to 1.37) for vermis hypoplasia and cerebellar hypoplasia, respectively. When the intercepts of the models (vermis hypoplasia: -1.02, *p* = 0.0005; cerebellar hypoplasia: -1.00, *p* = 0.0006) were back-transformed to the probability scale, the estimated ASD prevalence in studies with and without vermis hypoplasia was 35.2% and 26.5%, respectively. The estimated ASD prevalence in studies with and without cerebellar hypoplasia was 33.2% and 26.8%, respectively. Thus, studies that included vermis hypoplasia or cerebellar hypoplasia reported a higher ASD prevalence compared to those that did not. These differences were neither statistically significant nor clinically meaningful.

## Discussion

We estimated the prevalence of ASD in patients with CMs. Our meta-analysis of 32 articles revealed that 31.2% (or nearly 1 in 3) of individuals with CMs had ASD. The CDC’s most recent surveillance report estimated that 3.2% (1 in 31) of children in the United States have ASD [30].

A meta-regression revealed that the presence of vermis hypoplasia or cerebellar hypoplasia in a study did not predict differences in ASD prevalence. In contrast, the presence of Joubert syndrome in a study predicted differences in ASD prevalence. Studies that included Joubert syndrome reported 13.9% ASD prevalence, compared to 38.7% in studies without Joubert syndrome. This observation should be interpreted cautiously, given the inherent methodological limitations of study-level meta-regression. Meta-regression compares studies to studies, not individuals to individuals. As a result, we cannot determine if Joubert syndrome itself, rather than other unmeasured study-level differences, accounts for the lower ASD prevalence. These study-level differences may include the presence of multiple malformation types in a study, variable diagnostic methods for CMs or ASD, and the clinical profiles of recruited patients.

This work demonstrates that there is an association between CMs and high prevalence of ASD, amplifying the potential importance of close surveillance for autism in children with CMs. Many CMs are diagnosed prenatally [19]. Our results suggest that a child with a history of CM who presents to their pediatrician with concerns for developmental delays may benefit from a comprehensive neurodevelopmental assessment by a developmental neurologist or developmental-behavioral pediatrician. Pediatricians might consider a low threshold for referral in this population. Moreover, the Modified Checklist for Autism in Toddlers, Revised with Follow-Ups (M-CHAT-R/F) is a parent-report screening tool validated to assess ASD risk as early as 16 months of age [31]. This early screening and referral approach may benefit children with CMs threefold. First, early diagnosis and intervention improve neurodevelopmental outcomes in other populations of autistic children [20–23]. Second, early diagnosis decreases parental stress and anxiety [24]. Third, early intervention decreases overall disease burden and service utilization [25, 26]. Thus, proactive identification and intervention for autistic children with CMs may optimize developmental trajectories and reduce long-term costs.

This meta-analysis adds to a body of literature implicating the cerebellum in the pathophysiology of ASD [11, 12, 17]. The Diagnostic and Statistical Manual of Mental Disorders, Fifth Edition (DSM-5), defined ASD diagnostic criteria as persistent deficits in social communication and restricted, repetitive patterns of behavior [6]. The cerebellum influences these behaviors [1–3], raising the hypothesis that developmental changes to the cerebellum increase risk for ASD. Bolduc and Limperopoulos began to address this possibility previously [2]. They found that the prevalence of developmental/cognitive delay, language deficits, and social/behavioral deficits were increased in individuals with CMs. Here, we expanded on these results by estimating the prevalence of ASD in individuals with CMs. Our results support the idea that clinical diagnostic assessment for ASD can help children with CMs.

Our primary meta-analysis indicates developmental CMs are likely to increase risk for ASD. Preclinical tests of cerebellar function support this claim. Chemogenetic inhibition of Purkinje cells leads to social communication abnormalities and promotes restrictive, repetitive behaviors [32]. When Purkinje cell function was impaired in a *PTEN* knockout model of autism in mice, stereotyped repetitive behaviors increased in frequency [33]. These observations align with the clinical observation that Purkinje cells are decreased in number in ASD [17]. Our results support the notion that CMs may influence Purkinje cell function and increase ASD risk.

The results of our secondary meta-regression suggest that malformations of the midline vermis are associated with a higher prevalence of ASD. This observation is supported by evidence that the vermis is affected in ASD. The posterior vermis (lobules VI-X) is smaller in size in autistic individuals compared to neurotypical subjects [34–36]. Decreases in Purkinje cell number are observed in the vermis of mouse models of autism [37, 38]. Changes in function to the posterior vermis (e.g., lobules VI-VIII) contribute to differences in saccadic eye movements in autistic individuals [39]. Retrograde trans-neuronal transport of rabies virus in non-human primates revealed that this posterior area of the vermis receives projections from cingulate and primary motor cortices [40]. These regions could be implicated in the social and motor phenotypes of autism, respectively. Thus, developmental malformations of the vermis may impact integration of sensorimotor information that facilitates social interaction.

Our systematic review has at least five methodological limitations. First, most included studies are case series with small sample sizes. A small sample size increases the risk for type II error. A small sample size can also increase the risk of type I error by overestimating ASD prevalence. In the Forest Plot (Fig. 3), the wide confidence intervals of these small studies underscore the limited generalizability of the studies’ estimates. In contrast, studies with larger samples carried greater weight in the pooled analysis. Second, many studies lack a control group. Here, we compared the prevalence of ASD within each study to the general population. The prevalence of ASD varies across different countries and populations [41], so control groups may promote interpretability of our results. Third, studies use different metrics to diagnose ASD. During the study period, different versions of the DSM that handled ASD differently were published. Moreover, many studies only used clinician impression to diagnose ASD. These differences likely contribute high heterogeneity to our data. Moreover, these different metrics to diagnose ASD may result in under-identification of autism in earlier studies when different diagnostic criteria were used. Fourth, many studies recruited patients from hospitals and research centers, which limits the generalizability of the results to the general population while increasing the risk of selection bias. Fifth, included studies did not consistently test intelligence, adaptive functions, or other cognitive measures. As a result, we cannot draw conclusions about how co-morbid intellectual disability affects ASD prevalence in children with CMs.

Our study has at least one interpretive limitation. Some genetic variants that cause CMs also independently increase risk for ASD [33, 37, 42, 43]. For example, variants in *EN2*, *RELN*, *PTEN*, and *TSC1*/*TSC2* affect cerebellar development and have each been associated with ASD. In these cases, both the CM and ASD may reflect a single underlying disruption to brain development. This raises the possibility that a CM is one of several signs of that disruption, rather than a direct cause of ASD.

A prospective study that assesses frequency of co-morbid conditions and incorporates genetic testing, a psychometric ASD diagnostic tool, and a control group is needed. Such a study will determine whether genetic and/or medical factors account for the CM-ASD association, and more accurately quantify ASD prevalence in individuals with CMs.

## Conclusion

Our findings provide compelling evidence that ASD is more prevalent in individuals with CMs. The ability to detect CMs prenatally presents a critical opportunity to inform targeted autism screening protocols, enabling earlier diagnosis and intervention. We predict earlier intervention will improve developmental outcomes and reduce long-term healthcare burdens. A prospective multi-center study is needed to define the exact prevalence of ASD in each CM subtype. A recognition that cerebellar developmental anomalies contribute to ASD risk can transform clinical practice by enhancing diagnostic precision, accelerating access to services, and improving quality of life for affected children and their families.

## Supplementary Information

Supplementary Material 1.

## Data Availability

Data for this systematic review were extracted from published manuscripts.

## Footnotes

1 We use identity-first language (e.g., autistic individuals) throughout this manuscript, consistent with the preferences expressed by the autistic community and the recommendations for autism researchers [9, 10].
