# Supplementary material for: The prevalence of autism in cerebellar malformations: a systematic review and meta-analysis
Source: J Neurodev Disord. 2026 Apr 14;18:32. doi: 10.1186/s11689-026-09691-3 (PMC13224601; doi:10.1186/s11689-026-09691-3)
Supplement: Supplementary file 1 — Supplementary Material 1. [file 11689_2026_9691_MOESM1_ESM.docx]

**SUPPLEMENTAL INFORMATION**

**SUPPLEMENTAL INFORMATION TABLE 1**

| **PubMed** | |
| --- | --- |
| #1 | ("cerebellum/abnormalities"[MeSH Terms:noexp] OR "Agenesis of Cerebellar Vermis"[All Fields] OR joubert syndrome*[All Fields] OR dandy walker[All Fields] OR Gomez Lopez Hernandez[All Fields] OR rhombencephalosynapsis[All Fields] OR cerebellar hypoplasia[All Fields] OR pontocerebellar hypoplasia[All Fields] OR vermis hypoplasia[Title/Abstract] OR cranial fossa, posterior/abnormalities[MeSH Terms:noexp] OR "cerebellar malformation*"[Title/Abstract] OR "cerebellar agenesis"[Title/Abstract] OR "cerebellar dysgenesis"[Title/Abstract] OR "cerebellar heterotopia*"[Title/Abstract] OR "vermian hypoplasia"[Title/Abstract] OR "mega cisterna magna"[Title/Abstract]) |
| #2 | ("Autism Spectrum Disorder"[MeSH Terms] OR autis*[Title/Abstract] OR “Child Development Disorders, Pervasive"[MeSH Terms:noexp] OR "Attention Deficit and Disruptive Behavior Disorders"[MeSH Terms] OR attention deficit*[Title/Abstract] OR ADHD[Title/Abstract] OR hyperactiv*[Title/Abstract] OR "Child Behavior Disorders"[MeSH Terms] OR "Stereotypic Movement Disorder"[MeSH Terms] OR stereotypi*[Title/Abstract] OR "Language Development Disorders"[MeSH Terms] OR language[Title/Abstract] OR speech[Title/Abstract] OR neurodevelopmental[Title/Abstract] OR social skill*[Title/Abstract] OR "Developmental Disabilities"[MeSH Terms] OR "Aggression"[MeSH Terms:noexp] OR aggress*[Title/Abstract] OR neuropsycholog*[Title/Abstract] OR neuropsychiatr*[Title/Abstract] OR behavioral[Title/Abstract]) |
| #3 | #1 AND #2 |
| #4 | “English”[Language] |
| #5 | #3 AND #4 |
| #6 | "Humans"[MeSH Terms] |
| #7 | #5 AND #6 |
| #8 | "Animals"[MeSH Terms] OR "mice"[Title] OR "mouse"[Title] OR "rat"[Title] OR "rats"[Title] |
| #9 | #5 NOT #8 |
| #10 | #7 OR #9 |
|  |  |
| **Embase.com** | |
| #1 | ('autism'/exp OR 'attention deficit disorder'/de OR 'disruptive behavior'/exp OR 'impulse control disorder'/de OR 'abnormal behavior'/de OR 'automutilation'/de OR 'language disability'/exp OR 'communication disorder'/de OR 'behavior disorder'/de OR 'child behavior checklist'/de OR 'developmental disorder'/de OR 'developmental delay'/de OR 'hyperactivity'/de OR 'impulsiveness'/de OR 'compulsion'/de OR 'aggression'/de OR 'aggressiveness'/de OR 'anger'/exp OR 'social adaptation'/de OR 'social disability'/de OR 'social interaction'/de OR 'stereotypy'/de OR 'communication skill'/exp OR 'language ability'/de OR 'social behavior'/de OR 'behavior'/de OR 'neuropsychological test'/exp OR 'neuropsychology'/de OR 'neuropsychiatry'/de OR autis*:ti,ab,kw OR stereotyp*:ti,ab,kw OR social*:ti,ab,kw OR language:ti,ab,kw OR neuropsy*:ti,ab,kw OR behavioral:ti,ab,kw OR impulsiv*:ti,ab,kw) |
| #2 | ('joubert syndrome'/de OR 'rhombencephalosynapsis'/de OR 'dandy walker syndrome'/de OR 'gomez lopez hernandez syndrome'/de OR 'pontocerebellar hypoplasia'/de OR 'pontocerebellar hypoplasia type 1'/de OR 'pontocerebellar hypoplasia type 2'/de OR 'pontocerebellar hypoplasia type 6'/de OR 'cerebellum hypoplasia'/de OR 'cerebellar dysplasia'/de OR 'cerebellum agenesis'/de OR ('cerebellum vermis'/de AND 'hypoplasia'/mj) OR ('cerebellum'/de AND 'agenesis'/mj) OR 'dandy walker':ti,ab,kw OR joubert:ti,ab,kw OR 'gomez lopez hernandez':ti,ab,kw OR 'pontocerebellar hypoplasia':ti,ab,kw OR 'rhombencephalosynapsis':ti,ab,kw OR (cerebell* NEXT/1 (malformation* OR hypoplasia OR agenesis)) OR ((vermian OR vermis) NEXT/1 (hypoplasia OR agenesis)) OR 'mega cisterna magna':ti,ab,kw) |
| #3 | #1 AND #2 |
| #4 | english:la |
| #5 | #3 AND #4 |
| #6 | 'human'/de |
| #7 | #5 AND #6 |
| #8 | 'animal'/exp OR 'animal experiment'/exp OR 'nonhuman'/de OR mice:ti OR mouse:ti OR rat:ti OR rats:ti |
| #9 | #5 NOT #8 |
| #10 | #6 OR #9 |
|  |  |
| **APA PsycINFO** | |
| #1 | (joubert? adj3 syndrome*.mp. OR dandy walker.mp. OR Gomez Lopez Hernandez.mp. OR rhombencephalosynapsis.mp. OR cerebellar malformation*.mp. OR cerebellum malformation*.mp. OR cerebellar hypoplasia.mp. OR pontocerebellar hypoplasia.mp. OR vermian hypoplasia.mp. OR vermis hypoplasia.mp.) |
| #2 | (exp Autism Spectrum Disorders/ OR exp attention deficit disorder/ OR exp developmental disabilities/ OR "3250".cc. OR neurodevelopmental disorders/ OR neuropsychology/ OR neuropsychiatry/ OR exp mental disorders/ OR (autis* OR attention deficit* OR hyperactiv* OR self injur* OR language OR speech OR neurodevelopment* OR neuropsych* OR clinical OR mental illness*).ti,ab,id.) |
| #3 | #1 AND #2 |
| #4 | English.lg. |
| #5 | #3 AND #4 |
| #6 | Human.po. |
| #7 | #5 AND #6 |
| #8 | animal.po. or mice.ti. or mouse.ti. or rat.ti. or rats.ti. |
| #9 | #5 not #8 |
| #10 | #7 OR #9 |
| #11 | (0200 OR 0240 OR 0300 OR 0400).pt. |
| #12 | #10 not #12 |

**Supplemental Table 1: Search Strategies.**

**SUPPLEMENTAL INFORMATION TABLE 2**

| **CASE SERIES** | |
| --- | --- |
| **Number** | **Question** |
| 1 | Were there clear criteria for inclusion in the case series? |
| 2 | Was the condition measured in a standard, reliable way for all participants included in the case series? |
| 3 | Were valid methods used for identification of the condition for all participants included in the case series? |
| 4 | Did the case series have consecutive inclusion of participants? |
| 5 | Did the case series have complete inclusion of participants? |
| 6 | Was there clear reporting of the demographics of the partipants in the study? |
| 7 | Was there clear reporting of clinical information of the particpants |
| 8 | Were the outcomes of follow-up results of cases clearly reported? |
| 9 | Was there clear reporting of the presenting site(s)/clinic(s) demgraphic information? |
| 10 | Was the statistical analysis appropriate? |
|  |  |
| **CASE CONTROL STUDIES** | |
| **Number** | **Question** |
| 1 | Were the groups comparable other than the presence of disease in cases or the absence of disease in controls? |
| 2 | Were cases and control matched appropriately? |
| 3 | Were the same criteria used for identification of cases and controls? |
| 4 | Was exposure measured in a standard, valid, and reliable way? |
| 5 | Was exposure measured in the same way for cases and controls? |
| 6 | Were confounding factors identified? |
| 7 | Were strategies to deal with confounding factors stated? |
| 8 | Were outcomes assessed in a standard, valid, and reliable way for cases and controls? |
| 9 | Was the exposure period of interest long enough to be meaningful? |
| 10 | Was appropriate statistical analysis used? |
|  |  |
| **CROSS SECTIONAL STUDIES** | |
| **Number** | **Question** |
| 1 | Were the criteria for inclusion in the sample clearly defined? |
| 2 | Were the study subjects and the setting described in detail? |
| 3 | Was the exposure measured in a valid and reliable way? |
| 4 | Were objective, standard criteria usd for measurement of the condition? |
| 5 | Were confounding factors identified? |
| 6 | Were strategies to deal with confounding factors stated? |
| 7 | Were the outcomes measured in a valid and reliable way? |
| 8 | Was appropriate statistical analysis used? |

**Supplemental Information Table 2. Johanna Briggs Institute Critical Appraisal Checklist Questions.**
